# Maternal body mass index and placental weight: a role for fetal insulin, maternal insulin and leptin

**DOI:** 10.1007/s40618-022-01842-2

**Published:** 2022-07-04

**Authors:** O. Kristiansen, M. C. Roland, M. Zucknick, T. M. Reine, S. O. Kolset, T. Henriksen, T. Lekva, T. Michelsen

**Affiliations:** 1 Department of Obstetrics, Division of Obstetrics and Gynecology, Rikshospitalet, Oslo University Hospital, 0424 Oslo, Norway; 2 Institute of Clinical Medicine, Faculty of Medicine, University of Oslo, 0318 Oslo, Norway; 3 Institute of Basic Medical Sciences, Faculty of Medicine, University of Oslo, 0317 Oslo, Norway; 4 Norwegian Research Centre for Women’s Health, Rikshospitalet, Oslo University Hospital, 0424 Oslo, Norway; 5 Centre for Biostatistics and Epidemiology, Department of Biostatistics, Institute of Basic Medical Sciences, University of Oslo, 0317 Oslo, Norway; 6 Institute of Cancer Genetics and Informatics, Radiumhospitalet, Oslo University Hospital, 0424 Oslo, Norway; 7 Research Institute of Internal Medicine, Rikshospitalet, Oslo University Hospital, 0424 Oslo, Norway

**Keywords:** BMI, Placental weight, GWG, Adipokines, Insulin, Lipids

## Abstract

**Purpose:**

Placental weight (PW) has been found to mediate the main effect of maternal BMI on fetal size. Still, the BMI–PW association is poorly understood. Therefore, we aimed to explore potential explanatory variables, including gestational weight gain (GWG), early- and late-pregnancy circulating levels of maternal glucose, insulin, leptin, adiponectin, triglycerides, LDL-C, and HDL-C, and fetal insulin.

**Methods:**

We included two studies of pregnant women from Oslo University Hospital, Norway: the prospective STORK (*n* = 263) and the cross-sectional 4-vessel method study (4-vessel; *n* = 165). We used multiple linear regression for data analyses. A non-linear BMI-PW association was observed, which leveled off from BMI25. Therefore, BMI <25 and ≥25 were analyzed separately (*n* = 170/122 and 93/43 for STORK/4-vessel). Confounding variables included maternal age, parity, and gestational age.

**Results:**

PW increased significantly per kg m^−2^ only among BMI <25 (univariate model’s std.*β*[*p*] = 0.233 [0.002] vs. 0.074[0.48]/0.296[0.001] vs. −0.030[0.85] for BMI <25 vs. ≥25 in STORK/4-vessel). Maternal early- but not late-pregnancy insulin and term fetal insulin were associated with PW. The estimated effect of early pregnancy insulin was similar between the BMI groups but statistically significant only among BMI <25 (std.*β*[*p*] = 0.182[0.016] vs. 0.203[0.07] for BMI <25 vs. ≥25). Late pregnancy leptin was inversely associated with PW with a 1.3/1.7-fold greater effect among BMI ≥25 than BMI <25 in the STORK/4-vessel.

**Conclusions:**

The BMI–PW association was non-linear: an association was observed for BMI <25 but not for BMI ≥25. Leptin may be involved in the non-linear association through a placental–adipose tissue interplay. Maternal early pregnancy insulin and fetal insulin at term were associated with PW.

**Supplementary Information:**

The online version contains supplementary material available at 10.1007/s40618-022-01842-2.

## Introduction

In utero fetal growth and development are determinants of short- and long-term health [1, 2]. Maternal weight status, such as the pregestational body mass index (I), is a predominant determinant of birthweight, a commonly used indicator of fetal growth [3]. The epidemic increase in BMI throughout the last decades has led maternal pregestational overweight (BMI ≥25 <30 kg m^−2^) and obesity (BMI ≥30 kg m^−2^) to become major risk factors for adverse offspring health [4, 5]. However, the physiological factors explaining the association between maternal weight status and fetal growth are poorly understood, and insight into such factors would represent a new basis for early prevention of adverse health outcomes both from a short- and long-term perspective [5].

The placenta transports maternal nutrients to the fetus and regulates the fetal nutritional environment [6, 7]. Furthermore, the placental function is closely related to the placental weight (PW). First, PW is highly correlated with the villous surface area, which is the surface for transferring nutrients and oxygen [8]. Second, the placenta is an essential endocrine organ throughout pregnancy that secretes many hormones into the maternal blood circulation. These hormones cause profound changes in maternal metabolism, and maternal levels of some of these placental hormones are closely related to PW [9–11].

In a recent study [12], we found that PW mediates over 80% of the effect of pBMI and gestational weight gain (GWG) on birthweight after taking into account maternal glucose, insulin, leptin, and adiponectin, as well as fetal insulin and glucose uptake. Similarly, other previous studies have shown that adjustment for PW strongly reduces the estimated effect of maternal BMI on indicators of fetal growth [3, 13, 14]. The observed effects of PW on fetal growth call for studies of the determinants of placental growth. However, exploring potential factors in the association between BMI and PW is complicated because the placental hormones impact the same factors that potentially link maternal weight and PW. This challenges the hypotheses of a directed causal path between BMI and PW and necessitates an explorative approach.

The metabolic status among overweight women (BMI ≥25) differs from those with a normal pBMI (BMI <25). The metabolic changes accompanying overweight in a non-pregnant state include a relative insulin resistance accompanied by reduced glucose tolerance, higher insulin and leptin levels, lower adiponectin levels, and altered lipid profiles, especially high plasma triglycerides (TG) and low-density lipoprotein cholesterol (LDL-C) and lower high-density lipoproteincholesterol (HDL-C) [15–17]. Therefore, these and other BMI-related metabolic entities are potential factors linking maternal BMI to birthweight and PW.

GWG is independently associated with PW [14, 18]. However, the effect of GWG on birthweight partly depends on the pregestational BMI, i.e., a larger impact of GWG on birthweight has been observed among lean women than overweight women [19]. Hence, a similar moderating impact of the pregestational BMI could also occur for the association between GWG and PW. Furthermore, GWG is partly affected by placental hormones and the maternal pregestational metabolic state, which again is affected by the pregestational BMI. These interrelationships challenge a directed causal association [19].

The maternal factors may affect PW through their influence on substances in the fetoplacental circulation [20, 21]. Hence, the link between BMI and PW may include fetal factors. In previous publications, fetal insulin has been reported to be closely related to maternal glucose levels [12, 22, 23]. Furthermore, fetal insulin has been shown to stimulate villous endothelial proliferation, and thus promote placental growth [20]. Therefore, fetal signals may adjust the placental properties, including weight, transport systems and metabolism, to secure proper supply depending on the fetal needs. In particular, the fetal responses to a surplus of energy supply, as in the case of a high pregestational BMI, are still poorly understood.

In the current study, we aimed to explore a set of potential variables associating maternal BMI and PW on both the maternal and fetal sides of the placenta, including early- and late-pregnancy maternal circulating levels of insulin, glucose, leptin, adiponectin, triglyceride, LDL-C and HDL-C, and term pregnancy fetal levels of insulin.

## Methods

### Design and study population

Two separate and previously described study samples were included in the current study, the STORK cohort and the 4-vessel method study sample [3, 24]. Both studies were conducted at Oslo University Hospital, Rikshospitalet, and were approved by the Regional Committee for Medical and Health Research Ethics—South-East Norway (S-01191 and 13,885, respectively). All participants signed written informed consent.

The STORK study was a prospective cohort study with a longitudinal design including individuals who gave birth between 2002 and 2008 [3]. The current study included a subsample from the STORK cohort (*n* = 300 of 1031), previously described in detail with a flow chart for the inclusion of participants [25, 26]. This subsample of the STORK cohort was selected based on the detailed biochemical data available. In particular, data regarding adiponectin and leptin levels were available only in this subsample. Participants with missing biochemical variables were excluded (*n* = 17), leading to a final study sample of 263 individuals. Data from the first (1v) and last antenatal visits (4v) were included (14–16 and 36–38 weeks of gestation, respectively). The 4-vessel method study was a cross-sectional in vivo study of women scheduled for cesarean sections between 2012 and 2015. The current study included a subsample (*n* = 165 of 179), previously described, including flow charts for the inclusion of participants [12].

The STORK cohort included women of Scandinavian heritage with singleton pregnancies. Exclusion criteria were multiple pregnancies, pre-existing diabetes mellitus, fetal malformations discovered at routine ultrasound examination, and major maternal comorbidities. The 4-vessel method study included healthy, non-smoking women with uncomplicated singleton pregnancies. Exclusion criteria were significant pre-existing comorbidity, medication (other than levothyroxine and occasional use of antiallergics, antiemetics, antibiotics, and antacids), pregnancy complications, and the onset of labor before scheduled cesarean section.

### Clinical data and blood sampling

The blood samples from the STORK cohort were collected in the morning after an overnight fast. Maternal BMI (kg m^−2^) was calculated from the weight measured at the first antenatal visit using a calibrated scale and the measured maternal height. GWG was calculated as the difference between the weight measured at the first and last antenatal visit. Outcomes of pregnancy were collected from hospital records.

In the 4-vessel method study, the maternal characteristics were collected at enrollment, and pregnancy outcomes were obtained from hospital records. Maternal height and pregestational weight (*n* = 153, 93%) or first-trimester weight (*n* = 7) were obtained from antenatal health cards and were mainly self-reported. The risk of bias in self-reported data was considered. However, self-reported data obtained from antenatal health cards are regarded as acceptable as weight references [27]. Furthermore, analyses from the STORK study have indicated a very strong correlation between self-reported pregestational weight and the weight measured in v1 (*R*^2^ = 0.9) [28]. Therefore, we considered the data from the antenatal health cards to be reasonably reliable. On the day of delivery, the women were weighed on an impedance scale (Tanita Body Composition Analyzer, Tokyo, Japan). GWG was calculated as the difference between pre-pregnancy and term weight.

The 4-vessel sampling method is a unique and specialized blood sampling method previously described and illustrated [12]. Briefly, blood samples were collected during a planned cesarean section procedure, and the women were in a fasting state (>8 h). No glucose infusion was administered during the procedure. We collected blood samples from the maternal radial artery and uterine vein, and fetal umbilical vein and artery, representing blood vessels supplying and draining the placenta from both the maternal and fetal compartments. Cesarean section was performed under spinal anesthesia.

In both studies, gestational age at birth was based on ultrasound assessment of fetal biometric measures made at weeks 17–19. We dichotomized participants into nulliparous women for women who gave birth for the first time and multiparous for one or more previous births. The placentas were weighed untrimmed immediately after delivery.

### Biochemical parameters

Biochemical parameters were analyzed in venous serum for the STORK study. For the 4-vessel method study, arterial plasma was analyzed as it represents the blood to which the uteroplacental unit is exposed and, therefore, may be regarded as the most relevant for studying placental physiology. For both studies, glucose was analyzed at the accredited laboratory at Oslo University Hospital using the hexokinase method with reagents from Roche [24, 29, 30]. Insulin was assayed in duplicate (RIA, DPC, Los Angeles, CA, USA) in the STORK study [29] and at the accredited laboratory at the Department for Medical Biochemistry using the electrochemiluminescence immunoassay (Roche Diagnostics, Elecsys Insulin, 12,017,547 122) in the 4-vessel method study [24]. In both studies, lipids were analyzed at the accredited laboratory at the Department for Medical Biochemistry, Oslo University Hospital [31, 32]. However, in the STORK study, LDL-C was calculated using Friedewald's formula (LDL-C = total cholesterol−HDL-C−[triglyceride 5^–1^]), which correlates well with beta quantification of LDL-C (*r* = 0.937) [33]. Total adiponectin and leptin were measured in duplicate using commercially available reagents by enzyme immunoassay (EIA; R&D Systems, Minneapolis, MN) as described in a previous publication from STORK [34]. In the 4-vessel method study, the high molecular weight (HMW) adiponectin and leptin were analyzed in duplicates using a commercially available enzyme-linked immunoassay (ELISA) (HMW and total Adiponectin ELISA, ALPCO, Salem, NH, USA, 47-ADPHU-E01 and Quantikine ® ELISA, R&D systems ®, DLP00) as previously described [12].

### Statistics

Statistical analyses were conducted using SPSS (Version 27.0) [35].

Data are presented as median (25th and 75th percentiles, and min and max). Comparisons between women with BMI <25 and ≥25 were done using *t*-tests for Gaussian data. We used the *F*-test for comparing two variances to decide if the *t*-test that assumes equal variances (*p* > 0.05) or the test allowing unequal variances (*p* < 0.05) was used. Mann–Whitney-U tests were used for non-Gaussian data and Fisher’s Exact test for categorical data. The normality of the data was evaluated using graphical distribution plots (histograms and boxplots).

Missing data from the 4-vessel method study, including pregestational weight or first-trimester weight (*n* = 5), term weight (*n* = 3), PW (*n* = 1), and fetal insulin levels (*n* = 2), was handled by imputation using the R (version 3.6.3) package Multivariate Imputations by Chained Equations (MICE) (version 3.9) by predictive mean matching with *n* = 20 multiple imputations [36].

Using scatterplots, we observed a non-linear association between BMI and PW, i.e., the association between BMI and PW differed for women with BMI <25 and BMI ≥25. Therefore, the samples were stratified into corresponding BMI groups, and all analyses were conducted for the whole study sample, the BMI <25 and BMI ≥25-group, to evaluate differential effects between the groups. The assumptions for performing linear regression analyses were checked.

Multiple linear regression models with PW as the dependent variable were analyzed. A more detailed description of the regression analyses, including the different models, is supplied in the supplemental manuscript. Briefly, there were three final adjusted models (Adjusted models). Adjusted model 1, representing the effect of early pregnancy metabolic status, was based on data from the STORK study and included BMI, confounding variables (i.e., maternal age, gestational age at birth, and parity), and maternal early pregnancy levels (v1) of glucose, insulin, adipokines, and lipids. Adjusted model 2 represented late-pregnancy metabolic status and was also based on data from the STORK study and included BMI, confounding variables (as Adjusted model 1), GWG, and maternal late-pregnancy levels (v4) of glucose, insulin, adipokines, and lipids. Adjusted model 3 represented the late-pregnancy metabolic state and was based on data from the 4-vessel method study and included BMI, confounding variables (as Adjusted model 1), GWG and maternal term levels of glucose, insulin, adipokines, and lipids, and fetal levels of insulin.

## Results

### The study samples’ characteristics

The characteristics of the STORK and 4-vessel method samples are presented in Tables 1–4. PW was statistically significantly (*p* < 0.05) higher in the BMI ≥25 than BMI <25-groups in both studies (mean∆ [95% CI] = 48.7 g [1.9, 95.6] for the 4-vessel method study and 60.1 g [20.6, 99.7] for the STORK cohort). The two BMI groups differed significantly in most metabolic parameters, except maternal levels of LDL-C and fetal levels of insulin (Tables 3and 4). Compared to the BMI <25-group, the BMI ≥25-group of the STORK study early pregnancy (v1), late pregnancy (v4) and 4-vessel method study, respectively, had increased levels of glucose (mean∆ +4.3%, +6.0% and +4.9%), insulin (median∆ +68.2%, +103.2% and +45.1%), leptin (median∆ +97.1%, +67.1% and +52.4%), and triglycerides (median∆ +19.4%, +12.6%, and +21.0%). A lower level, on the other hand, was observed for adiponectin (median∆ −19.2%, −9.9% and −24.7%) and HDL-C (median∆ −11.6%, −10.5% and −14.1%).

**Table 1:** Clinical characteristics of the participants in the STORK cohort

	All individuals (*n* = 263)				BMI <25 (*n* = 170)				BMI ≥25 (*n* = 93)				
Characteristics	*N* (%)	Median	Q1–Q3	Min–Max	*N* (%)	Median	Q1–Q3	Min–Max	*N* (%)	Median	Q1–Q3	Min–Max	*P*_BMI <25 vs. BMI ≥25_
Maternal age, years	–	32	29–35	21–42	–	32	29–34	21–42	–	33	30–36	22–41	0.09^d^
Higher education, yes^a, b^	231 (88.2)	–	–	–	155 (91.2)	–	–	–	76 (82.6)	–	–	–	0.047^d^
Married/ partner, yes^a^	259 (98.9)	–	–	–	169 (99.4)	–	–	–	90 (97.8)	–	–	–	0.28^e^
Parity, nulliparous^a^	131 (50.0)	–	–	–	96 (56.5)	–	–	–	35 (38.0)	–	–	–	0.006^e^
Gestational age, weeks	–	40.4	39.3–41.1	36.7–42.6	–	40.3	39.1–41.0	36.7–42.6	–	40.6	39.7–41.4	37.4–42.6	0.056^d^
Smoking during pregnancy	8 (3.0)	–	–	–	4 (2.4)	–	–	–	4 (4.3)	–	–	–	0.46^e^
BMI, kg m^−2^	–	23.7	21.6–26.1	17.2–40.1	–	22.4	20.7–23.6	17.2–24.9	–	27.2	25.8–29.2	25.0–40.1	–
Gestational weight gain, kg	–	10.1	8.2–12.6	−0.8–22.9	–	10.0	8.4–12.4	0.3–22.9	–	10.4	8.0–13.0	−0.8–19.4	0.76^d^
Offspring sex, girl^a^	129 (49.2)	–	–	–	88 (51.8)	–	–	–	41 (44.6)	–	–	–	0.30^e^
Birthweight, g^a^	–	3658	3298–4012	2325–5090	–	3556	3228–3935	2325–4780	–	3780	3475–4230	2670–5090	<0.001^d^
Placental weight, g	–	700	600–840	370–1200	–	685	600–800	370–1150	–	750	645–900	430–1200	0.003^d^

^a^Descriptive statistic is missing for one individual (BMI ≥25)

^b^Higher education, ≥3-year education from college or university

^c^Smoking during pregnancy: 1 ≤ 10 cigarettes for 7 individuals and ≥10 for 1 individual (BMI ≥25)

^d^*t*-test (two tailed)

^e^Fisher’s exact test (two sided)

**Table 2:** Clinical characteristics of the participants in the 4-vessel sampling cohort

	All individuals (*n* = 165)				BMI <25 (*n* = 122)				BMI ≥25 (*n* = 43)				*P*_BMI <25 vs. BMI ≥25_
Characteristics	*N* (%)	Median	Q1–Q3	Min–Max	*N* (%)	Median	Q1–Q3	Min–Max	*N* (%)	Median	Q1–Q3	Min–Max	
Maternal age, years	–	36	33–38	27–44	–	36	32–38	27–44	–	36	33–39	30–44	0.46^b^
Higher education, yes^a^	144 (87.3)	–	–	–	110 (90.2)	–	–	–	34 (79.1)	–	–	–	0.07^c^
Employed, yes	158 (95.8)	–	–	–	118 (96.7)	–	–	–	40 (93.0)	–	–	–	0.38^c^
Married/ partner, yes	160 (97.0)	–	–	–	120 (98.4)	–	–	–	40 (93.0)	–	–	–	0.11^c^
Parity, nulliparous	41 (24.8)	–	–	–	32 (26.2)	–	–	–	9 (20.9)	–	–	–	0.54^c^
Gestational age, weeks	–	39.3	39.0–39.4	37.1–42.0	–	39.3	39.0–39.4	37.1–42.0	–	39.3	39.0–39.4	38.4–42.0	0.96^d^
In vitro fertilization, yes	13 (7.9)	–	–	–	12 (9.8)	–	–	–	1 (2.3)	–	–	–	0.19^c^
Smoking, non-smoker	158 (95.8)	–	–	–	118 (96.7)	–	–	–	40 (93.0)	–	–	–	0.38^c^
Smoking, stopped in 1st trimester	7 (4.2)	–	–	–	4 (3.3)	–	–	–	3 (7.0)	–	–	–	–
Gestational diabetes, yes	4 (2.4)	–	–	–	2 (1.6)	–	–	–	2 (4.7)	–	–	–	0.28^c^
Fasting, hours past midnight	–	10	9.0–11.0	8.5–17.5	–	10	9.0–11.0	8.5–17.5	–	10	9.0–11.5	8.5–15.0	0.89^d^
Pregestational body mass index, kg m^−2^	–	22.3	20.8–25.1	17.0–47.6	–	21.7	20.3–22.9	17.0–24.98	–	27.6	26.0–30.4	25.0–47.6	–
Gestational weight gain, kg	–	15.1	11.8–17.9	−1.2–31.0	–	15.3	12.5–18.0	4.7–31.0	–	14.9	11.3–17.8	−1.2–22.3	0.08^a^
Offspring sex, girl	73 (44.2)	–	–	–	55 (45.1)	–	–	–	18 (41.9)	–	–	–	0.86^c^
Birthweight, g	–	3558	3222–3826	2297–4955	–	3517	3196–3747	2297–4430	–	3662	3380–4005	2753–4955	0.006^b^
Placental weight , g	–	613	520–680	310–1115	–	612	515–676	310–989	–	617	544–739	396–1115	0.04^b^

^a^Higher education, ≥ 15-year education

^b^*t*-test (two sided)

^c^Fisher’s exact test (two sided)

^d^Mann–Whitney test (two sided)

**Table 3:** Biochemical characteristics of the participants in the STORK cohort

	All individuals (*n* = 263)			BMI <25 (*n* = 170)			BMI ≥25 (*n* = 93)			*P*_BMI <25 vs. BMI ≥25_
Characteristics	Median	Q1–Q3	Min–Max	Median	Q1–Q3	Min–Max	Median	Q1–Q3	Min–Max	
Visit 1 (Gestational week 14–16)										
Glucose, mmol L^−1^	4.6	4.4–4.8	3.3–5.4	4.5	4.3–4.8	3.3–5.4	4.7	4.5–5.0	3.8–5.4	<0.001^a^
Insulin, pmol L^−1^	27.0	18.0–38.0	8.0–160.0	22.0	15.0–31.0	8.0–66.0	37.0	28.5–53.0	13.0–160.0	<0.001^b^
Adiponectin, µg mL^−1^	8.8	6.8–10.6	2.5–27.7	9.2	7.5–11.1	3.0–27.7	7.5	5.8–9.5	2.5–24.3	<0.001^b^
Leptin, µg L^−1^	43.1	29.2–64.6	6.6–182.1	36.2	25.5–45.6	6.6–102.7	71.4	50.1–89.7	17.8–182.1	< 0.001^b^
Triglyceride, mmol L^−1^	1.0	0.9–1.3	0.1–2.8	1.0	0.8–1.2	0.1–2.4	1.2	1.0–1.5	0.6–2.8	<0.001^b^
LDL-C, mmol L^−1^	2.4	2.0–2.9	0.9–6.5	2.3	2.0–2.9	0.9–6.5	2.6	2.1–3.0	1.2–4.6	0.048^b^
HDL-C, mmol L^−1^	1.8	1.6–2.1	0.7–3.3	1.9	1.6–2.2	1.0–3.3	1.7	1.4–1.9	0.7–2.6	<0.001^a^
Visit 4 (gestational week 36–38)										
Glucose, mmol L^−1^	4.4	4.2–4.8	3.4–6.8	4.3	4.2–4.6	3.4–6.8	4.6	4.4–5.0	3.6–5.6	<0.001^a^
Insulin, pmol L^−1^	40.0	27.0–64.0	8.0–283.0	31.0	24.0–45.0	8.0–274.0	63.0	43.0–84.5	15.0–283.0	<0.001^b^
Adiponectin, µg mL^−1^	7.1	5.6–9.1	2.4–21.0	7.5	5.8–9.3	2.8–19.7	6.7	5.3–8.5	2.4–21.1	0.01^b^
Leptin, µg L^−1^	53.4	35.4–77.6	5.4–182.1	44.4	30.2–64.7	5.4–159.2	74.2	46.5–96.8	18.6–182.1	<0.001^b^
Triglyceride, mmol L^−1^	2.3	1.8–2.8	1.0–6.1	2.2	1.8–2.6	1.0–4.5	2.4	2.0–3.0	1.2–6.1	0.007^b^
LDL-C, mmol L^−1^	3.8	3.0–4.7	1.6–8.5	3.9	3.1–4.8	1.6–8.5	3.7	2.9–4.4	1.6–8.1	0.21^b^
HDL-C, mmol L^−1^	1.7	1.4–2.0	0.8–3.2	1.8	1.4–2.1	0.8–3.2	1.6	1.3–1.9	0.8–2.6	0.001^a^

^a^*t*-test (two sided)

^b^Mann–Whitney test (two sided)

*HDL-C* high-density lipoprotein cholesterol; *LDL-C* low-density lipoprotein cholesterol

**Table 4:** Biochemical characteristics of the participants in the 4-vessel sampling cohort

	All individuals (*n* = 165)			BMI <25 (*n* = 122)			BMI ≥25 (*n* = 43)			*P*_BMI <25 vs. ≥25_
Characteristic	Median	Q1–Q3	Min–Max	Median	Q1–Q3	Min–Max	Median	Q1–Q3	Min–Max	
Glucose, radial artery, mmol L^−1^	4.5	4.2–4.8	3.5–6.6	4.4	4.2–4.8	3.5–6.2	4.6	4.4–5.0	3.7–6.6	0.009^a^
Insulin, radial artery, pmol L^−1^	57.8	37.6–86.0	9.7–374.7	56.1	35.0–75.9	9.7–159.6	81.4	47.6–110.6	26.0–374.7	0.001^b^
Insulin, umbilical vein, pmol L^−1^	66.5	41.2–90.8	15.1–283.3	65.9	41.7–84.3	16.7–233.4	76.4	40.9–100.4	15.1–283.3	0.16^b^
Adiponectin, HMW, µg mL^−1^	2.7	1.9–3.6	0.3–17.6	3.1	1.9–3.8	0.3–17.6	2.3	1.8–2.8	0.5–5.8	0.004^b^
Leptin, µg L^−1^	20.3	11.7–28.7	3.7–78.2	17.9	10.4–24.2	3.7–71.0	27.3	19.4–39.0	11.8–78.2	<0.001^b^
Triglyceride, mmol•L^−1^	2.2	1.8–2.6	1.1–4.9	2.1	1.8–2.5	1.1–4.2	2.5	1.9–2.8	1.4–4.9	0.001^a^
LDL-C, mmol L^−1^	3.5	2.9–4.2	1.5–6.6	3.5	2.9–4.2	1.5–5.8	3.4	2.9–4.1	1.7–6.6	0.99^a^
HDL-C, mmol L^−1^	1.7	1.4–2.0	0.8–3.1	1.7	1.5–2.0	0.8–3.1	1.5	1.3–1.7	0.9–2.2	<0.001^a^

^a^*t*-test (two sided)

^b^Mann–Whitney test (two sided)

*HDL-C* high-density lipoprotein cholesterol; *LDL-C* low-density lipoprotein cholesterol

### The association between maternal BMI and PW

**Fig. 1:**
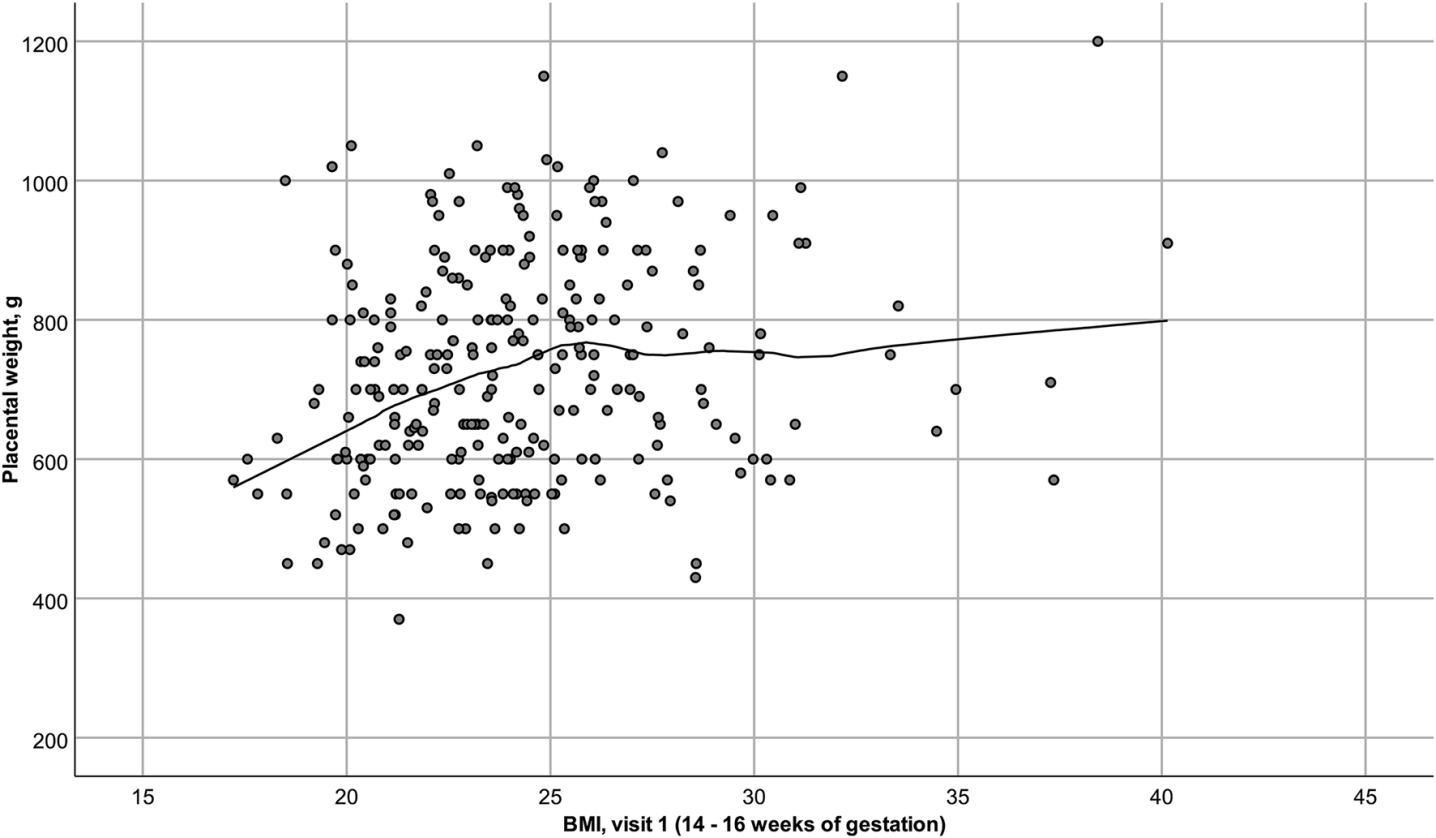
Scatterplot illustrating the association between BMI and PW for the STORK cohort. The figure was made using SPSS [35]

We observed a positive association between BMI and PW that leveled off at approximately BMI25 in both studies (Fig. 1 and Supplementary Fig. 1). In the stratified univariate linear regression analyses, the estimated standardized effect (standardized [std.] *β*) of BMI on PW was larger and only statistically significant in the BMI <25-groups (Supplementary table [Sup.Tab.] 1 and **2**).

#### The normal-weight group (BMI < 25 kg m^−2^)

Compared to the univariate models, relatively small changes were observed for the estimated standardized effects of BMI on PW in the Adjusted models (described in Statistics), and they remained statistically significant (*p* < 0.05) (Tables 5–7). GWG also had a statistically significant impact on PW in the adjusted models representing late pregnancy, and the estimated effect size (std. *β*) was similar to that of BMI (Tables 6and 7).

**Table 5:** Adjusted model 1, the multiple linear regression model representing the effect of early pregnancy maternal metabolic state on term placental weight

	All individuals (*n* = 263)			*R*^2^_adjusted_ = 0.132		BMI <25 (*n* = 170)			*R*^2^_adjusted_ = 0.189		BMI ≥25 (*n* = 93)			*R*^2^_adjusted_ = 0.069	
	Unstd	Std	Sig	95.0% CI for *β*		Unstd	Std	Sig	95.0% CI for *β*		Unstd	Std	Sig	95.0% CI for *β*	
	*β*	*β*		Lower	Upper	*β*	*β*		Lower	Upper	*β*	*β*		Lower	Upper
(Constant)	−784.4	0.000	0.020	−1443.6	−125.3	−1403.8	0.000	0.001	−2213.8	−593.9	−120.4	0.000	0.86	−1441.1	1200.3
BMI, v1 kg m^−2^	5.9	0.138	0.16	−2.4	14.2	16.8	0.193	0.02	2.9	30.8	0.52	0.010	0.94	−13.7	14.8
Gestational age. weeks	31.6	0.249	<0.001	17.0	46.2	40.0	0.330	<0.001	23.2	56.8	20.4	0.149	0.19	−10.4	51.1
Maternal age, years	0.5	0.013	0.84	−4.5	5.5	5.9	0.148	0.06	−0.2	12.0	−7.1	−0.182	0.12	−16.2	2.0
Parity, ≥1 vs. 0	54.0	0.172	0.009	13.4	94.6	31.2	0.101	0.20	−16.7	79.0	118.8	0.368	0.005	37.5	200.2
Glucose, mmol L^−1^	1.8	0.004	0.95	−49.8	53.3	−19.5	−0.045	0.53	−80.1	41.1	39.0	0.088	0.42	−56.2	134.2
Insulin, pmol L^−1^	1.4	0.176	0.01	0.3	2.5	2.2	0.182	0.02	0.4	4.0	1.3	0.203	0.07	−0.1	2.8
Adiponectin, µg mL^−1^	−0.001	−0.017	0.79	−0.006	0.005	−0.004	−0.086	0.25	−0.010	0.002	0.010	0.206	0.08	−0.001	0.022
Leptin, µg L^−1^	−0.4	−0.071	0.43	−1.4	0.6	−0.2	−0.019	0.82	−1.7	1.3	−0.7	−0.129	0.34	−2.2	0.8
TG, mmol L^−1^	−13.5	−0.032	0.66	−73.6	46.6	−42.4	−0.082	0.32	−126.2	41.3	−11.7	−0.033	0.81	−108.1	84.6
LDL-C, mmol L^−1^	13.1	0.062	0.34	−14.0	40.3	8.6	0.042	0.60	−23.6	40.7	24.9	0.111	0.35	−28.0	77.8
HDL-C, mmol L^−1^	5.4	0.014	0.83	−43.0	53.7	14.6	0.040	0.60	−39.5	68.7	−65.9	−0.147	0.19	−165.0	33.2

The model was based on data from the STORK cohort. The dependent variable was placental weight at term. Independent variables included early pregnancy (i.e., 1v, 14–16 weeks of gestation) maternal body mass index (BMI), confounding variables (i.e., maternal age, gestational age at birth, and parity), and maternal early pregnancy levels of glucose, insulin, adipokines (i.e., adiponectin and leptin), and lipids (i.e., triglycerides [TG], low-density lipoprotein cholesterol [LDL-C] and high-density lipoprotein cholesterol [HDL-C]). Biochemical factors were analyzed in venous serum

**Table 6:** Adjusted model 2, the multiple linear regression model representing the effect of late-pregnancy maternal metabolic state on term placental weight

	All individuals (*n* = 263)			*R*^2^_adjusted_ = 0.260		BMI <25 (*n* = 170)			*R*^2^_adjusted_ = 0.266		BMI ≥25 (*n* = 93)			R^2^_adjusted_ = 0.215	
	Unstd	Std	Sig	95.0% CI for B		Unstd	Std	Sig	95.0% CI for B		Unstd	Std	Sig	95.0% CI for B	
	*β*	*β*		Lower	Upper	*β*	*β*		Lower	Upper	*β*	*β*		Lower	Upper
(Constant)	−773.5	0.000	0.010	−1359.1	−188.0	−1301.8	0.000	0.001	−2039.6	−563.9	−453.8	0.000	0.45	−1646.7	739.0
BMI, kg m^−2^	15.2	0.355	<0.001	8.6	21.7	21.4	0.246	0.001	9.1	33.8	10.7	0.212	0.08	−1.4	22.8
Gestational age, weeks	23.0	0.181	0.001	9.2	36.9	32.1	0.265	<0.001	15.8	48.4	18.0	0.132	0.22	−10.8	46.9
Maternal age, years	−0.3	−0.007	0.90	−5.0	4.4	4.2	0.107	0.17	−1.8	10.3	−6.9	−0.177	0.10	−15.1	1.4
Parity, 1 vs. 0	31.5	0.100	0.10	−6.1	69.0	−2.6	−0.008	0.91	−48.2	43.0	118.1	0.366	0.003	40.9	195.3
GWG, Kg	12.3	0.267	<0.001	6.2	18.3	10.9	0.234	0.007	3.0	18.8	16.5	0.382	0.002	6.3	26.7
Glucose, mmol L^−1^	56.8	0.170	0.004	18.5	95.2	41.4	0.126	0.08	−5.3	88.2	70.3	0.189	0.06	−3.1	143.7
Insulin, pmol L^−1^	−0.09	−0.021	0.75	−0.6	0.4	−0.03	−0.006	0.94	−0.8	0.8	0.13	0.035	0.75	−0.7	0.9
Adiponectin, µg mL^−1^	−0.003	−0.060	0.30	−0.01	0.003	−0.003	−0.051	0.47	−0.01	0.005	−0.002	−0.043	0.69	−0.01	0.01
Leptin, µg L^−1^	−2.0	−0.407	<0.001	−2.8	−1.2	−2.1	−0.338	<0.001	−3.2	−0.9	−2.0	−0.429	0.002	−3.2	−0.7
TG, mmol L^−1^	−9.1	−0.045	0.46	−33.3	15.1	−14.2	−0.060	0.43	−49.4	21.0	−5.1	−0.030	0.78	−41.2	31.0
LDL-C, mmol L^−1^	13.2	0.102	0.08	−1.5	27.9	8.8	0.072	0.33	−9.2	26.9	29.0	0.218	0.04	0.8	57.3
HDL-C, mmol L^−1^	−38.0	−0.113	0.06	−77.9	2.0	−35.9	−0.114	0.14	−83.3	11.4	−59.2	−0.152	0.170	−144.3	25.9

The model was based on data from the STORK cohort. The dependent variable was term placental weight. Independent variables included early pregnancy (1v, i.e., 14–16 weeks of gestation) body mass index (BMI), confounding variables (i.e., maternal age, gestational age at birth, and parity), gestational weight gain (GWG), and maternal late-pregnancy (4v, i.e., 36–38 weeks of gestation) levels of glucose, insulin, adipokines (i.e., adiponectin and leptin), and lipids (i.e., triglycerides [TG], low-density lipoprotein cholesterol [LDL-C] and high-density lipoprotein cholesterol [HDL-C]). GWG was calculated as the weight change between 1v and 4v. Biochemical factors were analyzed in maternal peripheral venous serum

**Table 7:** Adjusted model 3, the final multiple linear regression representing the effect of late-pregnancy metabolic state on term placental weight

	All individuals (*n* = 165)			*R*^2^_adjusted_ = 0.355		BMI < 25 (*n* = 122)			*R*^2^_adjusted_ = 0.403		pBMI ≥ 25 (*n* = 43)			*R*^2^_adusted_ = 0.186	
	Unstd	Std	Sig	95% CI		Unstd	Std	Sig	95% CI		Unstd	Std	Sig	95% CI	
	*β*	*β*		Lower	Upper	*Β*	*β*		Lower	Upper	*β*	*β*		Lower	Upper
(Constant)	12.7	–	0.983	− 1159.2	1184.7	− 193.9	–	0.758	− 1437.6	1049.9	178.4	–	0.930	− 3919.8	4276.6
BMI (kg m − ^2^)	9.3	0.289	0.001	3.9	14.6	16.9	0.231	0.004	5.4	28.3	5.1	0.161	0.41	−7.4	17.6
Maternal age (years)	−1.0	−0.027	0.69	−5.7	3.8	−2.0	−0.058	0.47	−7.6	3.5	5.3	0.143	0.41	−7.8	18.4
Parity (≥ 1 vs. 0)	51.6	0.166	0.02	10.1	93.2	23.8	0.159	0.054	− 0.4	47.9	57.5	0.165	0.33	−61.4	176.3
Gestational age (weeks)	5.9	0.028	0.68	− 22.3	34.2	8.4	0.042	0.59	− 22.3	39.1	1.3	0.005	0.98	−92.2	94.9
GWG (kg)	11.0	0.377	< 0.001	6.9	15.2	11.8	0.390	< 0.001	7.2	16.3	5.5	0.201	0.32	−5.6	16.5
Glucose, maternal (mmol L^−1^)	−9.8	−0.034	0.66	− 53.9	34.2	− 36.4	− 0.124	0.15	− 86.5	13.7	6.4	0.022	0.92	−118.8	131.6
Insulin, maternal (pmol L^−1^)	−0.3	−0.108	0.22	−0.9	0.2	-0.1	-0.034	0.72	−1.0	0.7	−0.2	−0.080	0.73	−1.3	0.9
Adiponectin, maternal, µg mL^−1^	0.3	0.005	0.95	−9.1	9.8	2.9	0.049	0.55	−6.5	12.3	−30.5	−0.230	0.23	−81.1	20.1
Leptin, maternal µg L^−1^	−2.5	−0.272	< 0.001	−4.0	−1.1	−2.1	−0.187	0.03	−4.0	−0.3	−2.6	−0.312	0.13	−6.0	0.8
Insulin, fetal, pmol L^−1^	1.0	0.334	< 0.001	0.6	1.4	0.9	0.249	0.002	0.4	1.5	0.9	0.401	0.03	0.1	1.7
Triglyceride, maternal, mmol L^−1^	32.8	0.171	0.02	5.3	60.3	60.7	0.286	0.001	24.9	96.6	−1.7	−0.010	0.95	−59.4	56.0
LDL-C, maternal, mmol L^−1^	9.0	0.065	0.35	−9.9	28.0	−2.6	−0.019	0.81	−23.5	18.4	40.7	0.300	0.10	−8.3	89.7
HDL-C maternal, mmol L^−1^	−44.8	−0.138	0.06	−91.5	1.8	−9.3	−0.031	0.29	−60.5	41.9	−78.6	−0.165	0.29	−228.9	71.7

The model was based on data from the 4-vessel sampling cohort. The dependent variable was term placental weight. Independent variables included pregestational body mass index (BMI), confounding variables (i.e., maternal age, gestational age at birth, and parity), gestational weight gain (GWG) and maternal term levels of glucose, insulin, adipokines (i.e., adiponectin and leptin), and lipids (i.e., triglycerides [TG], low-density lipoprotein cholesterol [LDL-C] and high-density lipoprotein cholesterol [HDL-C], and fetal levels of insulin. GWG was calculated as the difference between term weight and pregestational weight. Maternal biochemical factors were analyzed in peripheral arterial plasma, and fetal insulin was analyzed in umbilical venous plasma

Maternal early pregnancy insulin was the only early pregnancy metabolic variable that had a statistically significant impact on PW, and the standardized effect size was similar to BMI (Table 5). In contrast, the estimated standardized effect of late-pregnancy maternal insulin on PW was small and not statistically significant (Sup. Tab. 1 and 2, and Tables 6and 7). However, at term, fetal insulin statistically significantly affected PW in the BMI <25-group, and the estimated standardized effect size was similar to that of BMI (Table 7).

In a univariate model, maternal glucose levels in late pregnancy had a statistically significant estimated effect on PW in the STORK’s BMI <25-group, and the estimated standardized effect size was similar to that of BMI (Sup. Tab 1). However, the std. *β* was majorly reduced (46.8%) and only borderline significant (*p* = 0.08) in the adjusted model (Table 6). In the 4-vessel method study’s BMI <25-group, maternal glucose did not affect PW (Sup. Tab. 2 and Table 7).

In the BMI <25-groups, maternal leptin in late pregnancy had a statistically significant (*p* < 0.05) inverse impact on PW, with an estimated standardized effect size which was $\pm$40% that of BMI (+37.4% in the STORK and −36.8% in the 4-vessel method study) (Tables 6 and 7). Including leptin in the final Adjusted model (Tables 6and 7) increased the standardized effect of BMI by 44% for the STORK and 19% for the 4-vessel method study (std. *β* [*p*] before including leptin was 0.170 [0.018] and 0.194 [0.015] for STORK and 4-vessel method study, respectively). Similarly, the standardized effect of GWG increased 3.44-fold for STORK and 7% for the 4-vessel method study (std. *β* [*p*] before including leptin was 0.068 [0.90] and 0.364 [*p* < 0.001], for STORK and 4-vessel method study, respectively). In other words, including leptin in the model amplified the estimated standardized effect of both BMI and GWG on PW, with a more pronounced impact in the STORK compared to the 4-vessel method study. Adiponectin was not associated with PW (Sup. Tab 1 and 2 and Tables 6and 7).

The estimated effect of HDL-C from late pregnancy on PW was statistically significant inverse in univariate models (Sup. Tab.1 and 2) and baseline models, including BMI and confounders (from STORK: *β* [95% CI] = −58.7 g per mmol L^−1^ [−102.9, −14.5], std. *β* = −0.186, *p* = 0.009; and from 4-vessel method study: *β* [95% CI] =  −57.4 g per mmol L^−1^ [−108.9, −5.9], std. *β* = −0.191, *p* = 0.029). However, upon adjusting for the other metabolically relevant variables, the standardized effect was reduced (38.7% for the STORK and 83.8% for the 4-vessel method study) and no longer statistically significant (Tables 6and 7). The estimated effect of maternal TG on PW in the BMI <25-group differed between the studies. In the STORK cohort, TG had no impact on PW (Table 6), whereas, in the 4-vessel method study, a statistically significant positive impact was observed (std. *β* [*p*] = 0.286 [0.001]) (Table 7).

### The overweight group (BMI ≥25 kg m^−2^)

Even though PW was statistically significantly higher in the BMI ≥25-groups compared to BMI <25 (Tables 1and 2), no additional increase in PW per kg m^−2^ was observed in the BMI ≥25-groups (Sup. Tab.1 and 2). The standardized effect of BMI in the model representing late-pregnancy metabolic state (Adjusted models 2 and 3) was increased compared to univariate models (Δstd. *β* = 0.138 for the STORK and 0.191 for the 4-vessel method study) but still not statistically significant (Tables 6and 7). The increased estimated effect was observed upon adjusting for leptin, as discussed below. Similar to the BMI <25-groups, GWG in the BMI ≥25-group had an impact on PW in the STORK (std. *β* [*p*] = 0.382 [0.002]) (Table 6), but not in the 4-vessel method study (std. *β* [*p*] = 0.201 [0.32]) (Table 5). Similarly, the GWG in the BMI ≥25-group of the STORK was comparable to the BMI <25-group, while in the 4-vessel method study, it was borderline lower in the BMI ≥25-group than the BMI <25-group. The estimated effect of GWG was also increased by leptin, as discussed below.

The impact of maternal insulin from early pregnancy on PW at term in the BMI ≥25-group was borderline significant (*p* = 0.07) (Table 5). Nevertheless, the estimated effect was not clearly different from the BMI <25-group. The standardized effect was similar to that of the BMI <25-group (Table 5). However, similar to the BMI <25-group, none of the other metabolic variables reflecting the early pregnancy metabolic state were associated with PW.

Similar to the BMI <25-group, maternal insulin in late pregnancy was not associated with PW in the BMI ≥25-group (Tables 6and 7). However, fetal insulin had a statistically significant impact (*p* = 0.032) on PW in the Adjusted model 3, and the standardized effect was 1.6-fold higher than in the BMI <25-group (Table 7).

The late-pregnancy glucose from the STORK’s BMI ≥ 25-group had a statistically significant impact on PW in the univariate models (*p* = 0.007) (Sup. Tab. 1). The standardized effect was 1.2-fold larger than in the BMI <25-group. The estimated effect was also statistically significant upon including BMI, confounders, GWG, insulin, and adipokines in the model (*β*[95% CI] = 80.9 g per mmol L^−1^ [8.1, 153.7], std. *β* = 0.216, *p* = 0.030). In the latter model, the standardized effect was 1.8-fold larger than that in the BMI < 25-group. However, including the maternal lipids in the model caused a small reduction in the estimated effect (Δstd. *β* = −12.5%), leaving it borderline significant in the Adjusted model 2 (*p* = 0.06) (Table 6). Still, the standardized effect was 1.5-fold larger than that of the BMI <25-group. In the 4-vessel method study, the estimated effect of glucose in the BMI ≥25-group was borderline significant in a model including BMI, confounders, GWG, glucose, and insulin (*β* [95% CI] = 104.9 g per mmol L^−1^ [−8.5, 218.2], std. *β* = 0.365, *p* = 0.069). However, upon adjusting for fetal insulin, the beta-value was reduced by nearly 50% (*β* [95% CI] = 57.0 g per mmol L^−1^ [−68.0, 182.1], std.*β* = 0.199, *p* = 0.360).

As in the BMI <25-group, late-pregnancy leptin had a statistically significant negative impact on PW in the STORK’s BMI ≥ 25-group (*p* = 0.002) (Table 6). The standardized effect was 1.3-fold larger than in the BMI <25-group. In the 4-vessel method study, the estimated effect was not statistically significant (*p* = 0.13); nevertheless, the standardized effect value was 1.7-fold larger than in the BMI < 25-group (Table 7). Additionally, the estimated effect of BMI in the STORK increased from no effect (std. *β* [*p*] = −0.006 [0.95]) to borderline significant (std. *β* [*p*] = 0.212 [0.083]). An increased standardized effect was also observed in the 4-vessel method study, although less pronounced (std. *β* [*p*] changed from 0.097 [0.62] to 0.161 [0.41]). Similarly, the standardized effect of GWG was increased 1.92-fold in the STORK and 2.01-fold in the 4-vessel method study (std. *β* [*p*] before including leptin was 0.199 [0.07] and 0.100 [0.61] in the STORK and 4-vessel method study, respectively). Due to power limitations, these observed moderating effects could not be statistically confirmed. Nevertheless, post hoc analyses further showed a statistically significantly lower increase in leptin from early to late pregnancy among overweight relative to normal-weight women (mean difference [95% CI] = 7.44 [1.97, 12.9], *p* = 0.008). Moreover, in both BMI groups, the increase in leptin from early to late pregnancy was statistically significantly lower among women with placentas that were ≥700 g than <700 g (mean difference [95% CI] = 14.5 µg L^−1^ [8.4, 20.5], *p* < 0.001 for BMI <25, and 12.6 µg L^−1^ [2.1, 23.1], *p* = 0.02 for BMI ≥25). A placenta of 700 g corresponds to the 50th percentile from Thompson et al. 2007 [37]. Similar to the BMI <25-group, maternal adiponectin from late pregnancy and term were not associated with PW in the BMI ≥25-group (Sup. Tab. 1 and 2, Tables 6and 7).

In the BMI ≥25-groups, none of the early or late lipid values were statistically significantly associated with PW except for LDL-C from late pregnancy in the STORK cohort (Table 6).

## Discussion

### The association between maternal BMI and PW

In line with previous studies [12, 14, 18, 38, 39], we found a positive association between BMI and PW, and PW was significantly higher in the overweight (BMI ≥25) relative to the normal-weight groups (BMI <25). However, PW only increased per BMI-unit among the normal-weight women, suggesting that BMI-related factors promoting placental growth do not act in a consistent dose–response manner across the whole BMI range. However, as opposed to our hypothesis, only a relatively small reduction of the estimated effect was observed upon including maternal metabolic variables, GWG, or fetal insulin, and it remained statistically significant in most of the models tested. This indicates that none of the included variables were significant mediators of the effect of BMI on PW. Still, in late pregnancy, fetal insulin and maternal leptin were associated with PW independent of BMI, and potential moderating effects were observed for late-pregnancy leptin.

### The effects of the metabolic variables among overweight versus the normal-weight women

#### Insulin

The overweight women had increased maternal levels of insulin, glucose, triglycerides, and leptin and lower levels of HDL-C and adiponectin compared to the normal-weight women, suggesting reduced insulin sensitivity in line with previous reports [15–17].

Regarding early pregnancy metabolic state, maternal insulin was the only included variable that was associated with PW. The estimated effect was statistically significant among the normal-weight and borderline significant among the overweight women. Although we observed a difference in the statistical significance of the estimated effect of insulin and found evidence of lower insulin sensitivity among the overweight relative to the normal-weight women, the estimated effect of insulin was similar between the BMI groups. Unlike the early pregnancy maternal insulin, the late-pregnancy maternal insulin had no estimated effect on PW.

The circulating fetal insulin levels at term in the overweight group were not significantly different from the normal-weight group despite the evidence of lower maternal insulin sensitivity and significantly higher maternal glucose levels, closely related to fetal insulin levels [12]. Nevertheless, the estimated effect of fetal insulin on PW was increased in the overweight group relative to the normal-weight (1.6-fold larger).

Taken together, we observed that maternal insulin from early—but not late—pregnancy and the fetal insulin at term was associated with PW. These findings are in line with previous studies reporting that maternal early pregnancy insulin impacts PW at term [40] and a shift in the placental insulin sensitivity from the maternal to the fetal side with advancing gestation [41, 42]. The latter may be caused by a change in the placental insulin receptor’s localization, levels, and function throughout pregnancy [41, 42]. Neither the estimated effect of maternal early pregnancy insulin nor fetal insulin at term appeared to be potential contributors to the non-linear association between BMI and PW.

#### Leptin

The early pregnancy leptin levels were not associated with PW at term in either BMI group, despite that leptin is regarded as an essential hormonal signal during the early stages of pregnancy [43–49]. However, in line with the previous reports [50, 51], we observed an inverse association between late-pregnancy leptin and PW in both BMI groups. The estimated effect of leptin was 1.3- and 1.7-fold larger among the overweight than the normal-weight in the STORK and 4-vessel cohort, respectively. Furthermore, including late-pregnancy leptin in the adjusted models had an impact on the estimated effect of BMI on PW, which was largest (evaluated as the percentage change) in the overweight group. Therefore, the effect of leptin could contribute to the non-linear association between BMI and PW. Moreover, in line with a previous report [17], post hoc analyses showed that overweight women had a significantly lower increase in leptin from early to late pregnancy. In the current study, we additionally found that, in both BMI groups, women with larger placentas (≥700 g) had a significantly lower increase in leptin throughout pregnancy relative to those with smaller placentas (<700 g). Our observations suggest that the rise in maternal leptin throughout pregnancy may be protective against placental overgrowth. Alternatively, the magnitude of the rise in leptin throughout pregnancy may be a biological marker predicting the risk of placental overgrowth.

Conclusions regarding the underlying mechanisms for the inverse association between maternal leptin and PW cannot be made based on the current study or previous studies. We suggest that there are at least two possible explanations. First, leptin could impact the nutrient supply to the fetoplacental unit, similarly to its weight-regulating function in non-pregnant individuals. This could occur via combined effects on the vascular system and maternal metabolism [46–48, 52, 53]. The effects of leptin could differentiate depending on the adipose tissue size and distribution, similar to insulin sensitivity [10, 17, 43, 54–57]. Alternatively, the association could reflect the effects of placental hormones [9–11], which facilitate maternal adipose tissue mobilization towards the term [10, 58] and thereby impact maternal leptin levels [59]. Beyond this, the placenta itself is a source of leptin; however, the placental contribution to the maternal circulation is estimated to be only 14% of the overall maternal leptin production [60]. Furthermore, previous publications have suggested no association between maternal and placental leptin [61, 62]. Therefore, it appears unlikely that placental leptin production causes the inverse association between maternal leptin and PW.

#### Glucose

The estimated effect of maternal glucose in late pregnancy on PW was increased among overweight relative to the normal-weight women. It was reduced upon adjusting for other metabolic variables and was not statistically significant in the Adjusted models. Notably, a reduction of nearly 50% was observed in the estimated effect of glucose on PW upon adjustment for fetal insulin in the BMI ≥25-group. Combined with the previously observed close association between maternal glucose and fetal insulin [12], these observations support the glucose steal hypothesis [63]. Fetal insulin is well recognized to promote fetal growth [12, 22]. However, our observations support the hypothesis that fetal insulin may also promote placental growth [20, 64].

#### GWG

We observed a positive association between GWG and PW, in line with previous reports [12, 14, 18]. In the STORK cohort, GWG was associated with PW in both BMI groups, while in the 4-vessel cohort, GWG was only associated with PW in the BMI <25-group. The discrepancy between the two cohorts may partly be explained by the observation that the GWG in the STORK was comparable between the overweight and normal-weight women, while in the 4-vessel cohort, the GWG was borderline significantly lower among the overweight women. Furthermore, it cannot be excluded that other discrepancies in characteristics between the cohorts could also have moderating effects, including variations in the proportions of nulliparous women.

However, late-pregnancy maternal leptin also appeared to potentially play a role in this discrepancy between the studies due to its impact on the estimated effect of GWG on PW. The estimated effect was increased in the overweight group in both studies, and the percentage increase was similar in both cohorts. However, the estimated impact before adjusting for leptin was larger in the STORK.

#### Lipids

Overall maternal lipids, including  TG, LDL-C and HDL-C, did not have any clear impact on PW, and they had little or no impact on the estimated effect of maternal BMI on PW. The latter indicates that maternal lipids were not important mediators in the association between maternal pBMI and PW.

### Strength and limitations

This study included two separate and independent human cohorts that allowed a longitudinal perspective and a detailed study of the maternal and fetal side of the placenta on the association between maternal weight status and PW.

The longitudinal STORK gives the opportunity to study the impact of factors from both early and late pregnancy. Pregestational weight was not available. It is possible that the weight gain in the first trimester is of importance for placental growth and function and may explain some of the discrepancies between the cohorts [65]. Furthermore, longitudinal assessments of placental growth were not available.

The 4-vessel cohort is an invasive human in vivo study, which allows detailed studies of both maternal and fetal variables [32, 66]. However, the cross-sectional design on term pregnant women precludes its validity for other stages of pregnancy. The pregestational weight in the antenatal health cards was mainly self-reported; therefore, there is a risk of reporting bias. However, self-reported weight data from antenatal health cards are regarded as acceptable as weight references [27].

Sample sizes were carefully taken into account when planning the analyses by the selection of variables and by restricting the complexity of the analyses. Nevertheless, we acknowledge that the number of variables is large relative to group size and that this affects the precision of the effect estimates. Therefore, we considered the coefficient of determination (*R*^2^) when analyzing the data because it is robust, e.g., against issues of multi-collinearity. In addition to the *β* [95% CI] and *p*-value, we also report the standardized effects (std. *β*) to allow a comparison of the relative effects between different variables.

## Conclusions

The association between BMI and PW was non-linear: an association was observed for BMI <25, but no additional increase in PW for BMI ≥25. Our analyses suggest that leptin may be involved in the non-linear association through a placental–adipose tissue interplay. Maternal early pregnancy insulin and fetal insulin at term were associated with PW, in line with a shift in placental insulin sensitivity from the maternal to the fetal side of the placenta.

## Supplementary Information

Below is the link to the electronic supplementary material.

Supplementary file1 (DOCX 165 KB)

## Abbreviations

BMI: Body mass index
GWG: Gestational weight gain
HDL-C: High-density lipoprotein cholesterol
LDL-C: Low-density lipoprotein cholesterol
PW: Placental weight
TG: Triglycerides
