# Supplementary material for: Maternal body mass index and placental weight: a role for fetal insulin, maternal insulin and leptin
Source: J Endocrinol Invest. 2022 Jul 4;45(11):2105–21. doi: 10.1007/s40618-022-01842-2 (PMC9525437; doi:10.1007/s40618-022-01842-2)
Supplement: Supplementary file 1 — Supplementary file1 (DOCX 165 KB) [file 40618_2022_1842_MOESM1_ESM.docx]

# Supplementary

## Supplementary manuscript

### Statistical methods

The assumptions for performing linear regression analyses were controlled. First, we investigated scatterplots and correlation analyses to evaluate if the associations between the independent variables and placenta weight were linear and to control for collinearity among the independent variables. If the correlation coefficient was >0.7, the independent variables were not included in the same model. Furthermore, we evaluated the distribution of the standardized residuals by examining the histogram of the standardized residuals and the normal predicted probability (P-P) plot. Finally, we controlled for homoscedasticity by examining the scatter plot of the predicted values and residuals.

Each of the independent variables was analyzed in univariate regression models with placental weight as the dependent variable. Furthermore, multiple linear regression models with placental weight as the dependent variable were analyzed starting with “baseline models”, one which included BMI and the confounders as independent variables and a second that additionally included GWG. Thereafter, each of the other independent variables (i.e., maternal levels of glucose, insulin, adipokines, and lipids, fetal levels of insulin (only for the 4-vessel cohort) were included one by one in the baseline models. This enabled the evaluation of the impact of each variable on the coefficient of determination (R^2^) and the effect estimate of BMI and gestational weight gain on placental weight. Variables from the STORK-cohort v1 were included in the first baseline model, and variables from v4 and the 4-vessel sampling cohort were included in the first and second baseline models. Furthermore, all independent variables were included in the final adjusted models as described in the main manuscript. All variables were included in the models as non-transformed and continuous variables, except for parity, which was dichotomized as described above.

## Supplementary figures

Supplementary figure 1. Scatterplot illustrating the association between BMI and placental weight for the 4-vessel method study (n=165). The figure was made in SPSS [1].


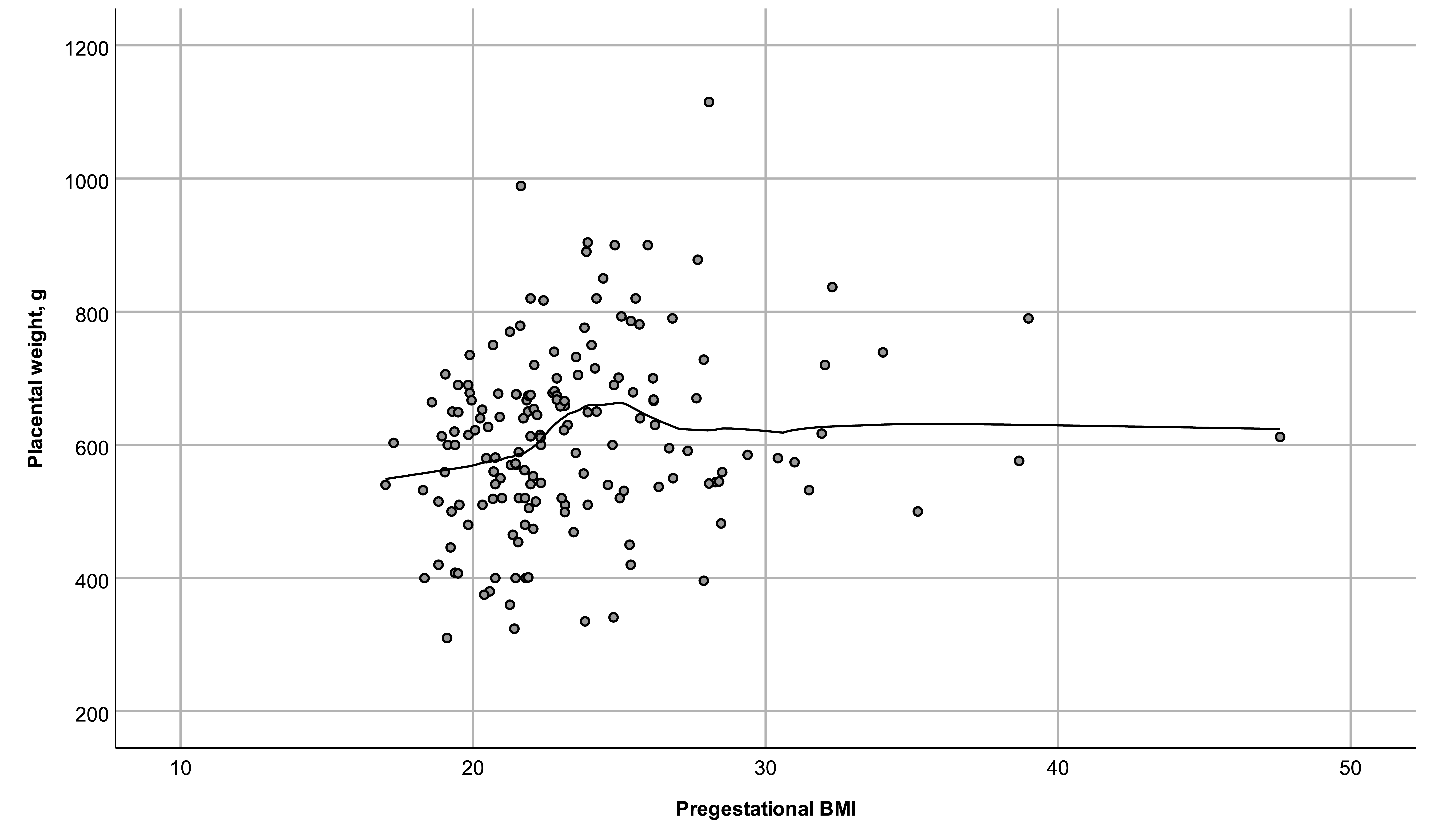


## Supplementary tables

Supplementary table 1 Univariate linear regression analyses with placental weight as the dependent variable based on data from the STORK cohort.

|  | All individuals (n=263) | | |  |  |  | pBMI<25 (n=170) | | |  |  |  | pBM≥25 (n=93) | | |  |  |  |
| --- | --- | --- | --- | --- | --- | --- | --- | --- | --- | --- | --- | --- | --- | --- | --- | --- | --- | --- |
|  |  | **Unstd.** | **Std.** | **p** | **95 % CI** | |  | **Unstd.** | **Std.** | **p** | **95 % CI** | |  | **Unstd.** | **Std.** | **p** | **95 % CI** | |
| Indep. var | **R^2^** | **β** | **β** |  | **Lower** | **Upper** | **R^2^** | **β** | **β** |  | **Lower** | **Upper** | **R^2^** | **β** | **β** |  | **Lower** | **Upper** |
| Mat. age,  years | 0.012 | 4.4 | 0.110 | 0.075 | -0.5 | 9.2 | 0.028 | 6.6 | 0.167 | 0.030 | 0.7 | 12.5 | 0.001 | -1.2 | -0.031 | 0.771 | -9.3 | 6.9 |
| Parity,  ≥1 vs. 0 | 0.042 | 64.0 | 0.204 | 0.001 | 26.6 | 101.5 | 0.026 | 49.7 | 0.161 | 0.04 | 3.4 | 96.1 | 0.043 | 67.1 | 0.208 | 0.045 | 1.4 | 132.8 |
| Gest. age, weeks | 0.068 | 33.2 | 0.261 | <0.001 | 18.3 | 48.2 | 0.116 | 41.4 | 0.341 | <0.001 | 24.0 | 58.8 | 0.003 | 6.9 | 0.050 | 0.63 | -21.5 | 35.2 |
| BMI, v1, kg m-^2^ | 0.056 | 10.1 | 0.236 | <0.001 | 5.0 | 15.1 | 0.054 | 20.3 | 0.233 | 0.002 | 7.4 | 33.2 | 0.005 | 3.7 | 0.074 | 0.48 | -6.7 | 14.2 |
| GWG, kg | 0.006 | 3.6 | 0.079 | 0.20 | -2.0 | 9.2 | 0.005 | 3.2 | 0.069 | 0.37 | -3.9 | 10.3 | 0.012 | 4.7 | 0.108 | 0.30 | -4.3 | 13.6 |
| Metabolically relevant variables from visit 1 (gestational week 14 – 16) | | | | | | | | |  |  |  |  |  |  |  |  |  |  |
| Glucose, mmol∙L^-1^ | 0.007 | 35.5 | 0.083 | 0.18 | -16.7 | 87.8 | 0.001 | -15.0 | -0.034 | 0.66 | -81.0 | 51.1 | 0.026 | 71.5 | 0.161 | 0.12 | -19.9 | 163.0 |
| Insulin, pmol∙L^-1^ | 0.056 | 1.9 | 0.236 | <0.001 | 1.0 | 2.9 | 0.036 | 2.3 | 0.190 | 0.01 | 0.5 | 4.1 | 0.031 | 1.2 | 0.177 | 0.09 | -0.2 | 2.5 |
| Adiponectin,  µg mL^-1^ | 0.008 | -0.004 | -0.090 | 0.15 | -0.009 | 0.001 | 0.010 | -0.004 | -0.099 | 0.20 | -0.010 | 0.002 | 0.003 | 0.003 | 0.058 | 0.58 | -0.007 | 0.013 |
| Leptin,  µg L^-1^ | 0.020 | 0.8 | 0.143 | 0.02 | 0.1 | 1.5 | 0.009 | 0.9 | 0.096 | 0.21 | -0.5 | 2.3 | 0.001 | -0.1 | -0.024 | 0.82 | -1.3 | 1.0 |
| TG,  mmol L^-1^ | 0.014 | 49.3 | 0.118 | 0.056 | -1.2 | 99.9 | 0.003 | 26.6 | 0.052 | 0.51 | -52.0 | 105.3 | 0.004 | 23.6 | 0.066 | 0.53 | -51.2 | 98.4 |
| LDL-C,  Mmol L^-1^ | 0.009 | 19.8 | 0.093 | 0.13 | -5.9 | 45.5 | 0.007 | 16.8 | 0.083 | 0.28 | -13.8 | 47.4 | 0.004 | 13.4 | 0.060 | 0.57 | -33.4 | 60.2 |
| HDL-C,  Mmol L^-1^ | 0.006 | -29.7 | -0.078 | 0.21 | -76.1 | 16.6 | <0.001 | 7.7 | 0.021 | 0.79 | -47.8 | 63.1 | 0.019 | -61.9 | -0.138 | 0.19 | -154.3 | 30.4 |
| Metabolically relevant models from visit 4 (gestational week 36 – 38) | | | | | | | | |  |  |  |  |  |  |  |  |  |  |
| Glucose, mmol L^-1^ | 0.083 | 96.4 | 0.288 | <0.001 | 57.3 | 135.4 | 0.056 | 77.7 | 0.237 | 0.002 | 29.2 | 126.2 | 0.078 | 103.7 | 0.279 | 0.007 | 29.3 | 178.1 |
| Insulin, pmol L^-1^ | 0.015 | 0.5 | 0.123 | 0.046 | 0.01 | 1.0 | 0.004 | 0.3 | 0.065 | 0.40 | -0.4 | 1.1 | 0.002 | 0.14 | 0.040 | 0.70 | -0.6 | 0.9 |
| Adiponectin, µg mL^-1^ | 0.018 | -0.01 | -0.132 | 0.03 | -0.013 | -0.001 | 0.013 | -0.01 | -0.113 | 0.14 | -0.014 | 0.002 | 0.012 | -0.01 | -0.107 | 0.31 | -0.016 | 0.005 |
| Leptin, µg L^-1^ | 0.008 | -0.5 | -0.092 | 0.14 | -1.1 | 0.2 | 0.042 | -1.3 | -0.205 | 0.007 | -2.2 | -0.3 | 0.030 | -0.8 | -0.173 | 0.098 | -1.7 | 0.2 |
| TG, mmol L^-1^ | 0.013 | 23.0 | 0.114 | 0.06 | -1.00 | 48.0 | 0.002 | 9.5 | 0.040 | 0.60 | -26.6 | 45.5 | 0.017 | 22.2 | 0.130 | 0.21 | -13.1 | 57.6 |
| LDL-C, mmol L^-1^ | 0.008 | 11.3 | 0.088 | 0.16 | -4.3 | 27.0 | 0.012 | 13.7 | 0.111 | 0.15 | -5.0 | 32.5 | 0.009 | 12.8 | 0.096 | 0.36 | -14.8 | 40.5 |
| HDL-C, mmol L^-1^ | 0.056 | -79.7 | -0.237 | <0.001 | -119.5 | -39.9 | 0.057 | -74.9 | -0.238 | 0.002 | -121.6 | -28.3 | 0.021 | -56.7 | -0.146 | 0.16 | -136.9 | 23.5 |

**Abbreviations:** BMI, body mass index calculated from the weight at visit 1 (gestational week 14 – 16); Gest. age., gestational age; GWG, gestational weight gain calculated by subtracting weight at visit 1 (gestational week 14 – 16) from the weight at visit 4 (gestational week 36 – 38); HDL-C, high-density lipoprotein cholesterol; Indep. var., independent variable; LDL-C, low-density lipoprotein cholesterol; TG, triglyceride.

Supplementary table 2 Univariate linear regression analyses with placental weight as the dependent variable based on data from the 4-vessel method study.

|  | All individuals (N=165) | | |  |  |  | BMI<25 (N=122) | |  |  |  |  | BMI≥25 (N=43) | | |  |  |  |
| --- | --- | --- | --- | --- | --- | --- | --- | --- | --- | --- | --- | --- | --- | --- | --- | --- | --- | --- |
|  |  | **Unstd.** | **Std.** | **p** | **95 % CI** | |  | **Unstd.** | **Std.** | **p** | **95 % CI** | |  | **Unstd.** | **Std.** | **p** | **95 % CI** | |
| Independent variable | **R^2^** | **β** | **β** |  | **Lower** | **Upper** | **R^2^** | **β** | **β** |  | **Lower** | **Upper** | **R^2^** | **β** | **β** |  | **Lower** | **Upper** |
| Mat. age,  years | 0.006 | 2.7 | 0.076 | 0.33 | -2.8 | 8.3 | 0.008 | 3.1 | 0.089 | 0.33 | -3.2 | 9.4 | <0.001 | 0.48 | 0.013 | 0.93 | -11.3 | 12.2 |
| Parity,  ≥1 vs. 0 | 0.074 | 85.0 | 0.273 | <0.001 | 38.7 | 131.4 | 0.067 | 38.6 | 0.259 | 0.004 | 12.6 | 64.7 | 0.050 | 78.2 | 0.225 | 0.15 | -28.8 | 185.3 |
| Gest. age,  weeks | <0.001 | 4.0 | 0.019 | 0.81 | -29.1 | 37.2 | 0.002 | 9.4 | 0.046 | 0.61 | -27.0 | 45.8 | 0.007 | -20.2 | -0.083 | 0.60 | -96.8 | 56.4 |
| pBMI,  kg m^-2^ | 0.040 | 6.4 | 0.201 | 0.010 | 1.6 | 11.3 | 0.088 | 21.6 | 0.296 | 0.001 | 9.0 | 34.3 | 0.001 | -1.0 | -0.030 | 0.85 | -11.0 | 9.1 |
| GWG,  kg | 0.079 | 8.2 | 0.280 | <0.001 | 3.9 | 12.5 | 0.166 | 12.3 | 0.408 | <0.001 | 7.3 | 17.3 | 0.009 | 2.6 | 0.097 | 0.53 | -5.9 | 11.2 |
| Glucose, rad. a., mmol L^-1^ | 0.008 | 26.5 | 0.092 | 0.24 | -18.0 | 71.0 | <0.001 | -6.4 | -0.022 | 0.81 | -59.4 | 46.5 | 0.065 | 73.4 | 0.256 | 0.098 | -14.1 | 160.8 |
| Insulin, rad. a.,  pmol L^-1^ | 0.003 | 0.2 | 0.054 | 0.49 | -0.3 | 0.7 | <0.001 | 0.02 | 0.006 | 0.95 | -0.8 | 0.8 | <0.001 | 0.01 | 0.003 | 0.98 | -0.7 | 0.7 |
| Insulin, umb. v.,  pmol L^-1^ | 0.087 | 0.9 | 0.295 | <0.001 | 0.4 | 1.3 | 0.057 | 0.9 | 0.239 | 0.008 | 0.2 | 1.5 | 0.117 | 0.8 | 0.342 | 0.03 | 0.1 | 1.4 |
| Adiponectin, rad. a., µg mL^-1^ | 0.009 | -6.4 | -0.095 | 0.22 | -16.8 | 3.9 | 0.002 | -2.3 | -0.040 | 0.67 | -13.1 | 8.4 | 0.051 | -29.9 | -0.226 | 0.15 | -70.7 | 10.8 |
| Leptin, rad. a.,  µg L^-1^ | 0.003 | -0.5 | -0.055 | 0.48 | 2.0 | 0.9 | 0.005 | -0.8 | -0.070 | 0.45 | -2.8 | 1.25 | 0.062 | -2.1 | -0.249 | 0.11 | -4.6 | 0.5 |
| TG, rad. a,  mmol L^-1^ | 0.091 | 58.0 | 0.302 | <0.001 | 29.7 | 86.4 | 0.180 | 90.1 | 0.424 | <0.001 | 55.3 | 124.9 | <0.001 | -1.5 | -0.009 | 0.96 | -55.1 | 52.1 |
| LDL-C, rad. a.,  mmol L^-1^ | 0.004 | 9.1 | 0.066 | 0.40 | -12.3 | 30.5 | <0.001 | -0.6 | -0.004 | 0.97 | -25.4 | 24.3 | 0.053 | 31.3 | 0.231 | 0.14 | -10.3 | 72.9 |
| HDL-C, rad.a.,  mmol L^-1^ | 0.080 | -91.7 | -0.282 | <0.001 | -139.9 | -43.5 | 0.066 | -77.2 | -0.256 | 0.004 | -129.8 | -24.5 | 0.068 | -124.3 | -0.260 | 0.09 | -269.7 | 21.1 |

**Abbreviations:** GWG, gestational weight gain; HDL-C, high-density lipoprotein cholesterol; LDL-C, low-density lipoprotein cholesterol; Mat. age., maternal age; rad. a., radial artery; umb. v., umbilical vein.

## References

1. IBM Corp (2020) IBM SPSS Statistics for Windows, Version 27.0. Armonk, NY: IBM Corp. <https://www.ibm.com/support/pages/downloading-ibm-spss-statistics-27>. Accessed 2020.
